# Zoonotic Pathogens among White-Tailed Deer, Northern Mexico, 2004–2009

**DOI:** 10.3201/eid1808.111902

**Published:** 2012-08

**Authors:** Citlaly Medrano, Mariana Boadella, Hugo Barrios, Antonio Cantú, Zeferino García, José de la Fuente, Christian Gortazar

**Affiliations:** Instituto de Investigación en Recursos Cinegéticos, Ciudad Real, Spain (C. Medrano, M. Boadella, J. de la Fuente, C. Gortazar);; Universidad Autónoma de Tamaulipas, Ciudad Victoria, Mexico (C. Medrano, H. Barrios);; Instituto Nacional de Investigaciones Forestales, Agrícolas, y Pecuarias, Tamaulipas, Mexico (A. Cantú);; Instituto Nacional de Investigaciones Forestales, Agrícolas, y Pecuarias, Morelos, Mexico (Z. García);; and Oklahoma State University, Stillwater, Oklahoma, USA (J. de la Fuente)

**Keywords:** zoonoses, viruses, epidemiology, tuberculosis, wildlife, transmission, Mycobacterium bovis, white-tailed deer, Odocoileus virginianus, host, vector, hepatitis E virus, tuberculosis and other mycobacteria, brucellosis, Brucella spp., raw deer meat, Mexico, United States

**To the Editor:** Intense wildlife management for hunting affects risks associated with zoonotic pathogens (*1*). White-tailed deer (*Odocoileus virginianus*) are increasingly managed by fencing, feeding, watering, and translocation to increase incomes from hunting in northern Mexico (*2*). These deer also play a major role in dissemination and reintroduction of pathogens and vectors from Mexico into the United States (*3**,**4*). White-tailed deer are suitable reservoir hosts for *Mycobacterium bovis* (*1*), and an *M. bovis*-positive white-tailed deer was recently found in Tamaulipas in northeastern Mexico (*2*). Brucellosis is widespread in many animal hosts in Latin America (*5*) and thus of interest in white-tailed deer. Another major zoonosis, sometimes linked to raw deer meat consumption, is hepatitis E, which is caused by genotypes of hepatitis E virus (HEV) (*6*). HEV is increasingly prevalent in red deer (*Cervus elaphus*) (*7*), but its prevalence in white-tailed deer is unknown.

The objective of this study was to determine the prevalence of zoonotic pathogens in white-tailed deer in northern Mexico. This study was conducted under a scientific collecting permit issued by the Mexican Division of Animal and Wildlife Health and on 8 ranches in 3 states in northern Mexico (≈26–28°N, 99–100°W).

Serum samples (n = 347) were collected during 2004–2009 in a cross-sectional survey for antibodies against HEV, *Brucella* spp., and mycobacteria. Deer were opportunistically sampled during live-capture operations as described by Cantú et al. (*8*). Bleeding was performed by using jugular venipuncture and vacuum tubes without anticoagulant. Samples were allowed to clot and centrifuged to collect serum that was stored at −20°C.

Serum samples were tested for IgG against HEV by ELISA as described (*7*). Serum samples were also tested for antibodies against *Brucella* spp. by using a commercial ELISA (Ingezim Brucella Compac 2.0 Ingenasa, Madrid, Spain), according to the manufacturer’s instructions. Detection of antibodies cross-reacting with 2 widely used mycobacterial antigens, bovine purified protein derivative (PPD) and paratuberculosis protoplasmatic antigen 3 (PPA3), was conducted as described (*9*). The sensitivity and specificity of this assay have not been established for white-tailed deer, but it has been used in seroprevalence studies of wild boar and fallow deer (*9**,**10*).

Insufficient volumes of serum samples prevented testing for antibodies against all pathogens (Table). Limited serum volume and lack of other (organ) samples also precluded additional analyses to verify presence of pathogens.

**Table:** Prevalence of serum antibodies against zoonotic pathogen antigens among white-tailed deer on 8 ranches, northern Mexico, 2004–2009*

Ranch no.	State	Municipality	Sample size	No. positive/no. tested (%)			
				HEV	*Brucella* spp.	Bovine PPD	PPA3
1	Tamaulipas	Guerrero	1	0/1 (0)	0/1 (0)	0/1 (0)	0/1 (0)
2	Tamaulipas	Guerrero	106	41/71 (57.7)	0/102 (0)	9/102 (8.8)	0/102 (0)
3	Tamaulipas	Nuevo Laredo	37	21/27 (77.7)	0	0/37 (0)	0/37 (0)
4	Nuevo León	Unknown	35	0	0	3/35 (8.5)	2/35 (5.7)
5	Coahuila	Guerrero	35	1/1 (100)	0/33 (0)	2/33 (6.0)	1/33 (3.0)
6	Coahuila	Guerrero	50	6/17 (35.2)	0/40 (0)	3/40 (7.5)	0/40 (0)
7	Coahuila	Hidalgo	17	4/6 (66.6)	1/16 (6.2)	3/16 (18.7)	5/16 (31.2)
8	Coahuila	Guerrero	66	16/19 (84.2)	0/39 (0)	7/39 (18.0)	0/39 (0)
NA	Total	NA	347	89/142 (62.7)	1/231 (0.4)	27/303 (8.9)	8/303 (2.6)

*HEV, hepatitis E virus; PPD, purified protein derivative; PPA3, paratuberculosis protoplasmatic antigen 3; NA, not applicable.

Prevalence was 62.7% (95% CI 54%–70%) for antibodies against HEV, 0.4% (95% CI 0%–2%) for antibodies against *Brucella* spp., 8.9% (95% CI 6%–13%) for antibodies against bovine PPD, and 2.6% (95% CI 1%–5%) for antibodies against PPA3 (Table). Antibody responses to bovine PPD were detected in deer from at 6 of 8 sampling sites; in deer from 3 of these sites, antibodies were also detected against PPA3 antigen. Seroprevalence against bovine PPD was higher than that against PPA3 (χ^2^ 10.9, df 1, p<0.01).

This cross-sectional survey of white-tailed deer in northern Mexico detected antibodies to several pathogens relevant to public and animal health. High prevalence of antibodies against HEV and frequent detection of antibodies against mycobacterial antigens are public health concerns. Prevalence of antibodies against HEV were 3× higher than that reported for red deer in Europe (*7*). This result suggests wide circulation of HEV in the study region and warrants further research, including detection and sequencing of virus RNA. Low *Brucella* spp. antibody prevalence confirms results of a study in this region (*8*).

Antibody responses to bovine PPD were detected in serum samples from deer from most sampling sites, occasionally in the absence of antibodies against PPA3. These results, and a recent report of an *M. bovis*–positive white-tailed deer from this region (*2*), suggest that these deer may be contracting *M. bovis* in northern Mexico. If one considers that white-tailed deer are *M. bovis* reservoirs in other parts of North America and that risk factors such as supplemental feeding are present in northern Mexico, there is a high risk for pathogen transmission to animals and humans (*1*). White-tailed deer are probably exposed to several human pathogens that are relevant to human and animal health in northern Mexico.

Although this cross-sectional survey provided only an indication of pathogen prevalence in the study populations, high antibody prevalence to HEV and mycobacterial antigens requires antigen-targeted surveillance. Risks associated with pathogen translocation by white-tailed deer are also relevant to neighboring states in Mexico and the United States.
